# Thoughts for Dentists

**Published:** 1859-12

**Authors:** Wm. A. Pease

**Affiliations:** Dayton, Ohio


### THOUGHTS FOR DENTISTS.

BY DR. WM. A. PEASE,
Of Dayton, Ohio.

Road at a Meeting of the Mad River Valley Dental Association, and published by its request.

GENTLEMEN: — The business of a Dentist is peculiar and distinct, in many important points of view, from that of any other profession, business or craft. The distinguishing characteristics are all adverse to his interests. They are so anomalous in character, that they can seldom be reached or corrected, by the ordinary rules that govern professional or commercial life; and the Dentist finds himself, therefore, isolated beyond the pale of the law and custom in many important particulars that are vital to him, while he himself is bound by all the rules and customs that govern professional life. This peculiarity of position is not only annoying; it is a positive evil, that subject him to frequent and serious losses, for which the law affords him no adequate remedy or redress; and, perhaps, what is equally injurious to him, it tends to destroy his confidence in the morality and honesty of man, if not to induce a certain laxity in himself. These evils can only be remedied by concert of action, or a generally recognized code of ethics. So long as the Dentist does not vindicate his own rights, but, however unwillingly, continues patiently to suffer these evils, he will find enough of those who will be willing he should, who will add, in an ironical tone, that a little suffering is good for the spirit.

Where there is a community of wants and interests, there must be a community of action; not only in reference to the government of the relations with the outward world, but also those of the individual members. So long as the Dentist acknowledges no higher code of law in his professional intercourse than that of Ishmael, he must not expect others to respect what he seems to be profoundly ignorant of himself, or what he holds in as profound contempt. Men respect those who habitually respect themselves; and, as there is a current opinion that the safety-valve of self-appreciation of each individual or profession, is set at the highest point it will bear consistently with safety, it cannot be expected that the community will have a very high opinion or appreciation of those who presistently depreciate the skill and the value of the services of each other, or who place so low a value on their own services as to rank them with those crafts where the remuneration is only measured by the wear and tear of muscle. Every profession should be idealized by each individual member thereof. In this respect, the law is especially worthy of commendation. The lawyer never asks, how much can I curtail my wants, that I may reduce my fees? but, how many high and noble wants can I create, that I may increase my fees in a like proportion? A lawyer never looks towards the minimum, but always towards the maximum fee, some distinguished man has obtained, and he crowds a point to make his equal that. Thus, there is an exhilerating professional atmosphere around its members, which strengthens, tones up, and develops powers and faculties to a point they otherwise would not attain. Besides, a lawyer seldom speaks disrespectfully of his brethren, or seeks to create a doubt as to their honesty or skill, as is too often done by physicians and dentists.

If such are the rules which govern a large, intellectual and powerful profession, whose chief business is to maintain the equalibrium of justice between man and man, how much more should similar rules be adopted by one to whose care so important interests, affecting, not only the purse, but the life, health, good looks; nay, the very individuality of members of community, are confided, without bonds or sureties, but simply on a reliance that its members are skillful, faithful, honest and honorable men?

The desire of dentists to have numerous calls—a bustling and crowded office, and the external appearance, at least, of a large and lucrative business, has fostered a disposition on the part of community to cheapen, and, perhaps, created a belief that the man who is satisfied with the smallest consideration for his services or skill, like the merchant or green grocer, is the one to be patronized. They seem to assume that skill, honor and integrity, like wares and marketable commodities, and that whoever has them for sale, at how low soever a price, has an article as similar to all others in the vicinity, as the goods in one store resemble those in another. For this, dentists are responsible. So long as they will continue to cheapen their labor to suit the importunities of their patients, they can make but little progress towards elevating the profession or themselves. It is now too often the case that a person starting for a dentist, goes as he would to purchase dry goods—to cheapen—and he will visit half a dozen offices before deciding.

Thus—A. B. Good morning, Doctor. I want a set of teeth. What is your lowest price for making one?

After a careful examination of the mouth, the price is named; and, if he is an honest and honorable man, it is such as he usually charges, and will afford him a fair and reasonable profit, and one he will not depart from to secure the set.

A. B.—Well, I am out this morning looking around. I'll look a little farther before I decide. If I can't do any better, I'll return. And away he goes to B. C.

A. B.—Doctor, I want a set of teeth. What is your lowest price for making one?

Another examination; the price is named, when A. B. exclaims:

That's high! Dr. —— will make them for less, (perhaps naming a smaller price than the Doctor actually fixed for the set.

Well, says Dr. B. C., I can make it as cheap as he can, and I will make it for so much; a still lower figure; and, besides, I will offer you this additional inducement. You have undoubtedly heard and read in the papers, that I have recently made several valuable discoveries; indeed, they are most marvelous; so much so, that the learned, able and conscientious editor of the Daily Puff-Well-For-Pay, came to see them, and he was so much pleased with them, that he has published a glowing account of them. Indeed, sir, it is my own opinion, and that also of my friends, that I will make a fortune from the sale of rights, and retire from business in a year. But, what I was going to offer you by way of inducement, and also to introduce it here, is a decided improvement. It is no less than an addition, or appendage, that I apply to all my sets of teeth. Why,sir, it is nothing less than a Patent Digestor, Chyle-Separator and Rejuvinator. It saves all the trouble of eating, one-half the food, and makes you grow younger every day—in fact, sir, I believe those who wear it will attain the very respectable age of Methuselah. They can't die—they can't die; they will have to be killed, or carried off bodily, to get out of the world! Besides, sir, I will make the set with my newly-invented and patented Double-Inflated, Double Safety-valved, Six-Horse-Power Engine. It always makes accurate fits, and never cracks the teeth!

But, A. B. thinks he will look for greater inducements elsewhere—he will see; and, true to his determination when starting from home, he employs the man who will work for the least money, and at the same time throw in the most Patent Digestors, &c.

Now, what is that man's opinion of dentists when he return home? Is it favorable—such as honorable men should desire? Is it not, that they are a set of sneaking, cheating Jews, who will cheat one for a farthing; and, furthermore, that they ought to be despised and kicked by every honest man?

It is high time these things should cease. It is high time dentists should learn to respect themselves and their patients, and, when consulted, state clearly, calmly and honestly what is required, and the exact price they demand for their services, and then refuse to depart from it. It is true, this course will seem odd at first—will be like a new life to many dentists and their patients. They will hardly know what to do with themselves, and their patients will open their eyes in mute astonishment—they won't know what to make of it; and when they get their things on, and get to the door, and the dentist still remains firm, don't fall a cent, don't offer to throw in any of his superior improvements—Patent Digestors, &c., &c.—they will begin to doubt his sanity. But, he need not expect that they will come back and order the set ef teeth from sheer admiration. No, no! not they! They will leave from mere force of habit and early education—they are bound to go the rounds this time. The Dentist has been too good a school-master; he has taught too thoroughly to have his teachings forgotten and disregarded so easily as this. Well, let them go; let others come and go in the same way; for so they will do; but don't flinch; keep a stiff upper lip, and bid them adieu as politely and calmly as if you had made your accustomed fee; for, remember, these persons are to be your best patients hereafter. You have lost them this time; you will loose others in the same way—you will think it is ruining you; that you are deserted, and have lost caste; but it is not so; if you have a strong back-bone, and you put your prices up instead of down, and stick to them; and, above all, don't offer to throw in, by way of inducmeent, any of your great improvements—Patent Digestors, &c.

After this period of probation is passed, you will see your profits represent your skill rather than your wasted purse, and you will begin to make money.

As a base for some further observations, I will here submit some propositions that should govern every office. I cannot see how a society can cohere without some such universally acknowledged rules among its members; viz:

Whenever a person calls to have teeth extracted, for an artificial denture, charge and collect at the time the price for extracting the teeth; say fifty cents a tooth.

Whenever a person calls to have a nerve destroyed, collect fifty cents for your service before he leaves the office.

Whenever a person calls for an artificial denture, whose mouth is already prepared, ask if the teeth were extracted by a neighboring dentist; if so, politely inform him you are not at liberty to set a price, or make it.

Whenever a person calls for an opinion as to the quality of a set of teeth, or the price he should have paid for it, unless glaringly defective, do not criticise it, or say he paid too much.

Never warrant a set of teeth, or a plug, for a day.

There are many other rules I would like to see universally adopted, but which may not seem equally as obvious to others. In regard to the first proposition, I wish to say, most dentists lose considerable sums every year; much larger than they think, unless they keep a record and examine it, by not adhering to this rule. All persons who have their teeth extracted and never return for the set of teeth, do not go to other dentists; some die, or leave the place; and others never have them inserted. Unless you have an open account with the person, ask for your fee before he leaves the office; and, if desirable, state to him the set of teeth will cost as much less when it is inserted.

The labor and cost of plugging a tooth where the nerve is destroyed, is so great, and the price is so low comparatively, though necessarily higher than other plugging, this method makes the fee seem more reasonable, as there is an open charge for two distinct services—besides many people never return to have the tooth plugged.

Lastly—Never warrant your operations. State distinctly to your patients that your charge is only a reasonable compensation for your present service; that if they break a tooth or a set, they wlll be charged a reasonable price for repairing it. Make your plugs stick, but do not warrant them to do so.

These propositions, for the government of the Dentists of this vicinity, are respectfully submitted to your consideration. If you see fit to modify, or adopt other and better ones, and also raise the price for dental operations, it will be gratifying to me, and I will say, in sporting phrase, "I will see you and go a little better."

